# Escaping Antiangiogenic Therapy: Strategies Employed by Cancer Cells

**DOI:** 10.3390/ijms17091489

**Published:** 2016-09-06

**Authors:** Mauricio P. Pinto, Paula Sotomayor, Gonzalo Carrasco-Avino, Alejandro H. Corvalan, Gareth I. Owen

**Affiliations:** 1 Department of Physiology, Faculty of Biological Sciences, Pontificia Universidad Católica de Chile, Santiago 8331150, Chile; gowen@bio.puc.cl; 2 Center for Integrative Medicine and Innovative Science, Facultad de Medicina, Universidad Andrés Bello, Santiago 8370071, Chile; paulacsf@yahoo.com; 3 Department of Pathology, Faculty of Medicine, Universidad de Chile, Santiago 8380456, Chile; gcarrasa@me.com; 4 Department of Hematology-Oncology, Faculty of Medicine, Pontificia Universidad Católica de Chile, Santiago 8330032, Chile; corvalan@med.puc.cl; 5 Center UC Investigation in Oncology (CITO), Pontificia Universidad Católica de Chile, Santiago 8330023, Chile; 6 Biomedical Research Consortium of Chile, Santiago 8331150, Chile; 7 Millennium Institute on Immunology & Immunotherapy, Santiago 8331150, Chile; 8 Advanced Center for Chronic Diseases (ACCDiS), Universidad de Chile, Santiago 8380492, Chile

**Keywords:** vasculogenic mimicry, vascular co-option, cancer dormancy, residual disease

## Abstract

Tumor angiogenesis is widely recognized as one of the “hallmarks of cancer”. Consequently, during the last decades the development and testing of commercial angiogenic inhibitors has been a central focus for both basic and clinical cancer research. While antiangiogenic drugs are now incorporated into standard clinical practice, as with all cancer therapies, tumors can eventually become resistant by employing a variety of strategies to receive nutrients and oxygen in the event of therapeutic assault. Herein, we concentrate and review in detail three of the principal mechanisms of antiangiogenic therapy escape: (1) upregulation of compensatory/alternative pathways for angiogenesis; (2) vasculogenic mimicry; and (3) vessel co-option. We suggest that an understanding of how a cancer cell adapts to antiangiogenic therapy may also parallel the mechanisms employed in the bourgeoning tumor and isolated metastatic cells delivering responsible for residual disease. Finally, we speculate on strategies to adapt antiangiogenic therapy for future clinical uses.

## 1. Tumor Angiogenesis and Antiangiogenesis

In 1939, Ide et al. [1] made the first observation that tumor growth was accompanied by the formation of new blood vessels, a process known as angiogenesis. They postulated that tumors could produce a “vessel-growth stimulating substance” and consequently these pioneering studies of almost 80 years ago are now considered the first investigations in the field of “tumor angiogenesis”. Over the following three decades, various studies using different models confirmed these observations and, gave rise the report of a “vasoproliferative effect” of transplanted malignancies [2,3,4]. In the 1960s, Folkman et al. published the first studies demonstrating that neovascularization was required for tumor growth. He observed that when cancer cells were injected into isolated perfused organs they remained as small tumors (<1–2 mm in size), however, if these tumors were transplanted into syngeneic mice they rapidly became vascularized and grew beyond a few millimeters [5]. In 1971, Folkman postulated that tumor growth was angiogenesis dependent and therefore the inhibition of this process could be therapeutic [6]. Thus the term “antiangiogenesis” was born, meaning a blockade of the formation of new blood vessels in a growing tumor. The concept developed by Folkman has been extensively confirmed by experimental and clinical studies. Tumor angiogenesis is now considered one of the “hallmarks of cancer” [7] and consequently over the last decades, the development of angiogenic inhibitors with therapeutic potential has been a central focus of cancer research.

The main molecular drivers of angiogenesis are members of the vascular endothelial growth factor (VEGF) family and their receptors. This family includes VEGF-A (commonly and hereafter named simply VEGF), VEGF-B, VEGF-C, VEGF-D, and placental growth factor (PIGF)-1 and -2. The VEGF-A gene can be spliced into multiple isoforms [8,9] but the most relevant for tumor angiogenesis are the soluble isoforms VEGF121 [10] and VEGF165 [8]. The main signaling receptor is VEGF receptor 2 (VEGFR2; also known as kinase insert domain receptor (KDR) in humans), a tyrosine kinase receptor (TKR) that is expressed in almost all endothelial cells (ECs) and binds VEGF, VEGF-C, VEGF-D, and VEGF-E. Through ligand activation, VEGFR2 has been shown to regulate EC proliferation, migration, and survival. The other two receptors are VEGFR1 (also known as FMS-like Tyrosine kinase 1 (FLT1)) that under activation promotes recruitment of endothelial progenitor cells and VEGFR3 (or FLT3) that is expressed almost exclusively in lymphatic endothelium. VEGFR2 and VEGFR3 can also function as TKRs. VEGF and its cognate receptors play a critical role in EC survival and angiogenesis [11]. One of the first commercially successful angiogenesis inhibitors to be developed was bevacizumab [12], a humanized monoclonal antibody against VEGF-165, the most predominant VEGF-A isoform. In 2004, bevacizumab became the first angiogenesis inhibitor approved by the Food and Drug Administration (FDA) in the United States. Today, a variety of compounds with angiostatic activity, including antibodies against VEGF and TKR inhibitors targeting VEGFR, have been approved for clinical use. Some of the FDA approved angiogenic inhibitors available for cancer treatment are listed in Table 1. These drugs can be used either as a single agent or as an adjuvant with chemotherapeutics. Currently, there are >800 active clinical trials evaluating angiogenesis inhibitors (https://clinicaltrials.gov/). Although many antiangiogenic therapies have been incorporated into standard protocol for certain cancer types, in some cases, approval has been withdrawn due to either lack of efficacy or concerns with toxicity [13]. A number of clinical studies that used angiogenesis inhibitors as adjuvants have failed to provide significant benefits to patients: a phase III trial assessed the effects of bevacizumab as an adjuvant in stage II–III colon cancer patients and did not meet its primary endpoint of prolonging the three-year disease-free survival (DFS) [14], another phase III randomized study in colon cancer also used bevacizumab in combination with different chemotherapy regimes and found no significant improvements in DFS and even suggests a detrimental effect in overall survival [15]. Additionally, in metastatic HER2-negative breast cancer patients the combination of bevacizumab with paclitaxel (PTX) improves DFS compared to PTX alone, however patients did not show significant differences in quality of life assessments [16]. Hence, despite the clinical benefits observed with FDA-approved antiangiogenic drugs, the limited outcome in patients has led to the notion that cancer cells can develop strategies to escape angiogenesis inhibition and become resistant.

## 2. Mechanisms for Escape/Resistance to Antiangiogenic Therapy

**Figure 1:**
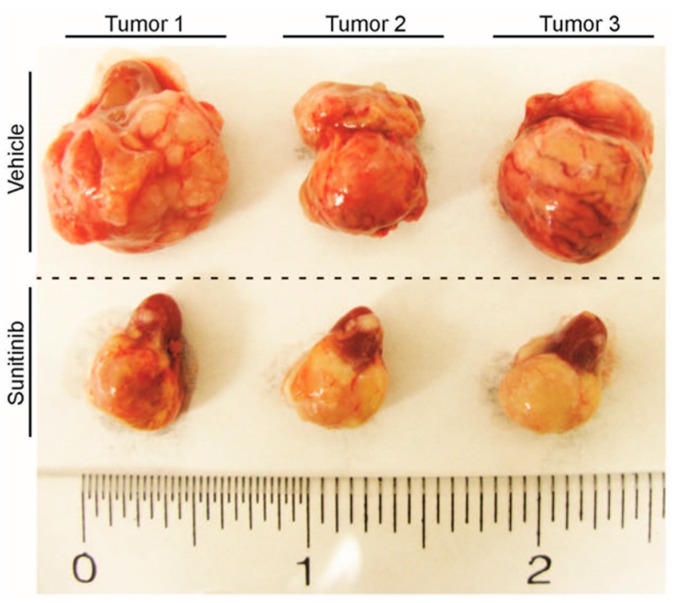
Antiangiogenic resistant tumors. Murine renal cancer tumors were generated by orthotopic injection of RENCA cells in Balb/C mice. Displayed tumors were obtained from mice treated with vehicle (distilled water with 1.8% NaCl, 0.5% carboxymethylcellulose, 0.4% tween 80 and 0.9% benzylalcohol, pH: 6.0) or Sunitinib (40 mg/kg, five days a week) for 17 days. Tumors were established for 10 days before treatment. All animal protocols were approved by the institutional bioethics committee at Universidad Andres Bello, Santiago, Chile. Approval #024/2014 (26 December 2014).

Cancer cells are defined by their heterogeneity and genetic instability [17], evolutionary biology teaches us that a selection pressure triggers reactionary dynamics and natural selection will produce a resilient system. Hence, antiangiogenic therapy or namely anti-VEGF therapy works upon cancer cells as a selection pressure and the surviving cells acquire an advantage in the new environment. An example of antiangiogenic compound used in the clinic is sunitinib, a small-molecule multi-targeted TKR inhibitor (TKI). Sunitinib is a FDA approved drug for renal cell carcinoma and for Imatinib (IM)-resistant (another TKI) gastrointestinal stromal tumors (GISTs). Figure 1 shows a sunitinib treated murine renal cancer (RENCA), where antiangiogenic treatment leads to noticeably less vascularized tumors. The resulting hypoxic tumor microenvironment is composed by normal fibroblasts, immune and ECs among others, which may play a major role in tumor growth and progression. Therefore, another potential mechanism of resistance to antiangiogenic therapies may result from growth signals provided by the microenvironment [18,19,20].

The methods of escape are numerous and may include: vascular heterogeneity, influence of the cancer stroma, stress adaptation, and alternative vascularization methods such glomeruloid angiogenesis, looping angiogenesis and intussusceptive growth [21,22]. In this mini-review, we will focus and discuss in detail recent advances on three main mechanisms of antiangiogenic therapy escape: upregulation of compensatory pathways, the process of vasculogenic mimicry (VM) and the concept of vessel (or vascular) co-option (VCO).

## 3. Upregulation of Alternative/Compensatory Pathways

Angiogenesis is an exquisitely complex [23] and critical process for both normal and tumor cells; consequently, one of the most common mechanisms employed by cancer cells to escape inhibition/blockade is the upregulation of redundant pathways.

In pancreatic cancer models, a VEGFR2 blocking antibody prevents tumor growth for approximately two weeks, however after this period tumors rapidly regrow and increase their vascularity with a concomitant increase in fibroblasts growth factor-1 (FGF-1), FGF-2, Ephrin-A1 (Eph-A1), Eph-A2 and angiopoietin-1 (Angpt-1) levels [24]. Clinically, colon cancer and glioblastoma patients increase their plasma FGF-2 levels following anti-VEGF therapy using bevacizumab or a VEGFR inhibitor respectively [25]. A number of studies have demonstrated crosstalk between VEGF and FGF-2 [26]; both factors can act synergistically to induce angiogenesis [27] and FGF-2 induced angiogenesis is partly dependent on VEGF activation [28]. Preclinical and clinical studies have evaluated FGF-2 targeting in cancer as an alternative to VEGF using small molecules [29,30,31] and monoclonal antibodies [32,33]. Recently, a small soluble decoy receptor fusion protein that comprises a truncated portion of the extracellular region of FGF Receptor-1 (named FGF-Trap) has demonstrated high affinity for FGF-2 and a potent inhibitory activity on the FGF signaling pathway, suppressing FGF-2 induced cell proliferation and significantly decreasing tumor growth and angiogenesis in vivo [34]. A clinical trial that included bevacizumab as an adjuvant in colorectal cancer reported increases in plasma levels of FGF-2, hepatocyte growth factor (HGF), placental growth factor (PIGF), stromal-derived factor (SDF)-1, and macrophage chemoattractant protein-3 [35]. In vivo, sunitinib-resistant tumors display increased HGF levels and ECs within these tumors overexpress c-*met* (the HGF receptor) [36]. In these animals, a combination of sunitinib and a selective c-*met* inhibitor significantly reduced tumor growth in sunitinib resistant tumors compared to either treatment alone, and a systemic injection of HGF in sensitive tumors conferred sunitinib resistance [36]. Aberrant c-*met* signaling has been reported in a variety of human cancers and clinical trials that incorporate selective drugs against c-*met* are ongoing [37].

The placental growth factor (PIGF) was initially discovered and cloned from human placenta where it plays a fundamental role in embryonic development. PIGFs (there are four isoforms) belong to the VEGF family [38], however their role in angiogenesis is somewhat controversial. In animal studies PIGF overexpression correlates with a decrease in tumor growth by stabilization of the tumor vasculature caused by heterodimerization with VEGF, that neutralizes its potency [39,40]. In contrast, PIGF in vitro is chemotactic for ECs and macrophages, mobilizes bone marrow-derived cells and increases VEGF-induced survival, proliferation and migration of ECs [41,42]. Applying a similar strategy to the one described for the FGF-trap, a VEGF-trap protein, called Aflibercept was produced by fusing the VEGF-binding domain of VEGFR1/VEGFR2 with the Fc portion of the human IgG1. Aflibercept (Table 1) acts as a high-affinity binding decoy receptor that neutralizes both VEGF and PIGF [43]. A phase III randomized trial that used Aflibercept plus a combination of fluorouracil, leucovorin and irinotecan (called FOLFIRI) found a significant increase in overall and progression free survival relative to placebo plus FOLFIRI in metastatic colorectal cancer (mCRC) patients that had been previously treated with oxaliplatin, the effects were also significant in patients that were previously treated with bevacizumab [44]. Currently, at least nine clinical trials that use Aflibercept are recruiting patients [45,46,47].

The platelet-derived growth factor (PDGF) pathway is another compensatory pathway usually upregulated in anti-VEGF treated tumors [48], PDGF ligands provide mitogenic signals, critical for pericyte recruitment and maturation. Studies demonstrate that PDGF is produced by endothelial and tumor cells [49], but can also be expressed by other cell types within the tumor microenvironment including carcinoma- or tumor-associated fibroblasts (CAFs or TAFs, respectively). In fact, TAFs derived from anti-VEGF resistant tumors upregulate their PDGF levels, and these TAFs also stimulate the growth of anti-VEGF sensitive cells under VEGF inhibition [50]. Studies also demonstrate that PDGF expressed by stromal fibroblasts is responsible for the increase in proliferation and angiogenesis in breast cancer cells in vitro [51]. As stated above, IM is a Tyrosine kinase inhibitor (TKI) drug originally developed as an inhibitor for the BCR-ABL kinase, a fusion protein that causes chronic myeloid leukemia (CML), however IM also targets PDGF Receptors. Studies demonstrate that IM can decrease angiogenesis both in vitro [51] and in vivo [52]; ECs can be activated to increase angiogenesis via a VEGF-independent mechanism through Neuropilin-1 (NRP-1), a non-catalytic receptor for VEGF165 that potentiates signal transduction of activated VEGFR2 [53,54], however NRP-1 is able to promote angiogenesis through a VEGF/VEGFR2 independent pathway by association with the Abelson murine leukemia viral oncogene homolog (ABL) kinase (a non-receptor kinase) and subsequent phosphorylation of Paxillin (PXN) and actin remodeling, a mechanism that can be inhibited by IM [55]. The same authors propose the use of IM to improve antiangiogenic therapies [55]. Accordingly, phase II/III studies evaluating the efficacy of IM as a single agent or as an adjuvant are currently under way [56,57].

Angiopoietins are a family of ligands that bind to the EC membrane receptor tyrosine kinase TIE2 (also known as TEK), Angpt-1 and Angpt-2 can activate TIE2 at different affinities. Alongside VEGF, the Angpt pathway plays a major role in tumor angiogenesis [58] and current late-stage clinical trials are targeting Angpt-1, Angpt-2 and TIE2 as a strategy against tumor growth [59]. Inhibition of this pathway may be applicable together with other treatments and applied to improve the efficacy of anti-VEGF therapy.

Delta-like ligand-4 (Dll-4) is a ligand for Notch expressed on the surface of arterial ECs [60]. Dll-4 and Notch are upregulated by VEGF and under physiological conditions act as a negative feedback mechanism for vessel sprouting and angiogenesis [61]. In cancer, Dll-4-Notch signaling pathway regulates tumor growth by decreasing angiogenesis, despite improving vascular function. Conversely, inhibition of Dll-4-Notch increases non-functional vasculature and reduces tumor growth [62,63,64]. Interestingly, preclinical and clinical findings have highlighted Dll-4-Notch signaling as a pathway involved in antiangiogenic resistance, specifically to anti-VEGF therapy [61,65]. Phase I and II clinical studies using Dll-4 inhibitors are currently ongoing [66].

In addition to the upregulation of proangiogenic factors, antiangiogenic therapy-induced hypoxia is able to trigger the recruitment of bone marrow-derived cells (BMDCs) [18]. Proangiogenic BMDCs are vascular progenitor cells that are recruited in part through increases in Hypoxia-Induced Factor 1α (HIF1α) and its downstream effector stromal-cell derived factor 1α (SDF1α) [67,68]. Therefore SDF1α could be exploited as a biomarker of antiangiogenic-treated relapsing tumors [18].

Another mechanism of antiangiogenic therapy resistance involves an increase in pericyte coverage. Inhibition of VEGF signaling can lead to a substantial reduction in microvascular density (MVD), however, functional vessels that remain after treatment are slim and tightly covered with pericytes [69]. Both in vitro and in vivo studies demonstrate that pericytes regulate EC proliferation and can promote EC survival through autocrine mechanisms [70,71]. Moreover TKI-based therapies that target both ECs via VEGFR inhibition and pericytes via PDGFR inhibition have higher therapeutic benefit compared to VEGF inhibitors alone.

## 4. Vasculogenic Mimicry (VM)

In many ways, a bourgeoning tumor, which has cells proliferating at a faster rate than the arrival of vasculature, is similar to a tumor undergoing antiangiogenic therapy. In both instances, the tumor needs a non-endothelial means of obtaining nutrients while disposing of cellular waste. One of the mechanisms behind this survival is speculated to be VM [72,73]; the term describes the formation of tubular structures within a tumor that are formed by cancer cells and independent of ECs [73].

These remodeled cancer cells are believed to secrete matrix proteins, such as collagens IV and VI, heparan sulfate proteoglycan and laminin, forming a tubular structure that is the reverse to traditional blood vasculature, where the basal lamina sits behind the ECs. Together with the aforementioned adopted expression of EC markers, polysaccharides are markers of VM and tubular structures are strongly positive for periodic acid-schiff (PAS) stain [74]. Now reported in 15 solid tumor types [75], this process was first described in uveal melanoma, the plasticity of cancer cells allows remodeling and acquisition of EC markers (such as CD31 and vascular endothelial (VE)-cadherin), and the formation of de novo vascular-like networks that may connect the tumor to the endothelial-lined vasculature [73]. These intratumoral structures provide a perfusion pathway transporting fluid from poorly formed/leaky tumor endothelial vasculature to the core of the malignant mass. Some authors have subdivided VM into two distinct forms: firstly, a tubular type that bares uncanny resemblance to blood vessels; and, secondly, a disjointed patterned matrix type that does not morphologically or topologically resembles physiological blood vasculature [74].

As is true for all malignant processes, VM may well be a physiological process hijacked by tumor cells. During pregnancy, the advance of the trophoblast into maternal uterine walls is speculated to involve the formation of non-endothelial vessel-like structures prior to the formation of the placenta [76]. Once adopted by either the bourgeoning tumor or metastatic foci, VM may be a way of controlling glucose and other nutrient supply while providing a drainage system for cellular secretions that eventually merges with endothelial-derived vasculature. Indeed, several authors have speculated on the presence of a “mosaic vasculature”, a fusion of both endothelial and non-endothelial derived vessels [77,78,79].

Currently, there is no consensus on how VM is activated in cancer cells. It is speculated that hypoxia, caused by the decline in blood vessel irrigation, following antiangiogenic treatment could be a trigger for VM [73]. In cancer cells, hypoxia brings about a change in cell phenotype and induces endothelial-to mesenchymal transition (EMT) [80], a phenotype believed to be linked to VM [81]. Although highly plausible, this theory remains controversial as many of the supporting in vitro studies showing HIF1α and hypoxia-related proteins may be involved are performed in cell cultures that may not represent VM [82].

**Figure 2:**
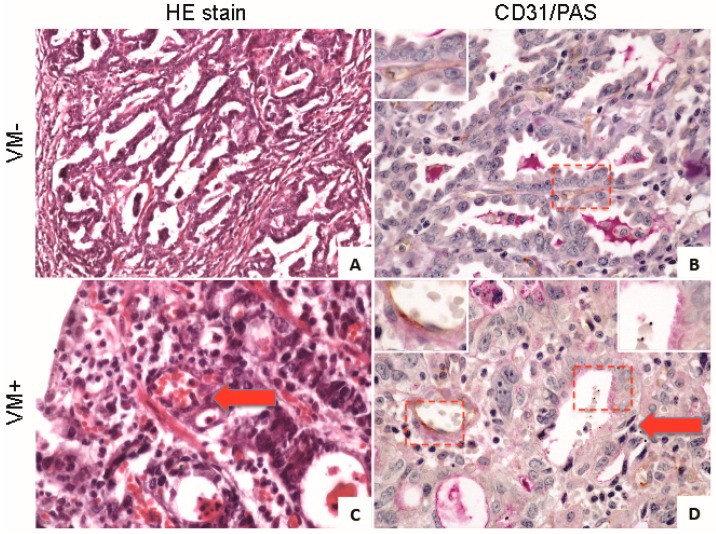
Vasculogenic mimicry in gastric adenocarcinomas: (**A**,**B**) Gastric adenocarcinoma sample negative for vasculogenic mimicry (VM), stained with hematoxylin and eosin (HE; Panel **A**, magnification 200×) or with a dual stain for the endothelial cell marker CD31/PAS (600×); Inset in Panel **B** (upper left) shows a CD31+ (brown) vessel between two glands; (**C**,**D**) VM+ adenocarcinoma sample. Panel **C** (600×) shows an HE stain, red arrow shows a vessel that contains red blood cells in its lumen; Panel **D**, CD31/PAS stain (600×) demonstrates two types of vessels. Upper left inset shows a CD31+ (brown, EC marker) vessel. Red arrows highlight a tumor gland CD31− (no brown) PAS+ that contains red blood cells in its lumen. Upper right inset shows a magnification of PAS+/CD31− vessel (VM+). Red frames in panels **B** and **D** indicate the area magnified in the respective insets.

In a meta-analysis of 22 clinical studies, encompassing 3062 patients across 15 cancer types, the five-year overall survival of VM+ and VM− cancer patients was 31% and 56%, respectively. Accordingly, the calculated relative risk of failure to achieve five-year survival in VM+ patients was significantly higher than that of VM− [83]. Interestingly, in all cancer types analyzed to date, the presence of VM is reported in fewer than 50% of cases and may thus be representative of a common aggressive malignant sub-class or phenotype. In osteoblastic-type osteosarcomas, the presence of VM was detected in 22.7% (15 of 66) of tumors. This incidence negatively correlated with both metastasis-free and overall survival, but was independent of patient sex, age, surgical type or tumor size [84]. Two histological analyses demonstrated the same percentage on VM formation in ovarian cancer. One study reported 52 (43%) out of 120 carcinomas analyzed were VM+ [85]. A previous study reported 43% (36 of 84) of analyzed ovarian carcinomas were VM+, within this group VM+ patients displayed significantly elevated levels of pathologic grade, histological type and poor survival [86]. Similarly, VM was detected in 35% of triple-negative breast cancers (TNBC) as opposed to 17.8% in non-TNBCs. In gastric adenocarcinoma, tubular networks were observed in 40 of 173 (22%) patients, with statistically elevated correlation with poorly differentiated tumors [87]. Figure 2 shows gastric adenocarcinoma samples from a negative (VM−, Figure 2A,C) and a positive (VM+, Figure 2B,D) patient for tubular networks stained with hematoxylin and eosin (HE; Figure 2A,C) and with a dual CD31 (brown)/PAS (violet) stain to identify EC vessels. Red arrow in Figure 2C shows a vessel that contains red blood cells in its lumen. In Figure 2D, red arrow highlights a CD31− vessel within a tumor gland that contains red blood cells, representing VM+ vessels seen in detail in the upper right inset, in contrast upper left inset shows a traditional (CD31+) used as a control. VM has also been identified in rhabdomyosarcomas, prostatic carcinomas, soft tissue sarcomas, glioblastomas, liver carcinomas and osteosarcomas among many other malignancies [88]. Thus, conventional therapeutic regimens that target angiogenesis alone may in the long-term be ineffective against a high percentage of invasive tumors capable of undergoing VM and thus presenting heterogeneous microcirculation.

Currently, there is only a limited understanding of the cellular basis of VM; non-endothelial tubular-structures can be obtained in vitro using aggressive cancer cell lines seeded on matrigel (aqueous constituents of the extracellular matrix proteins, obtained from a mouse sarcoma) or collagen I [89]. Authors report this phenomenon occurs in two forms; as quickly forming tubular structures reminiscing vessels formed within hours in classic EC tubular assays or as thicker multicellular structures that form after several days in culture [82]. While the claim that these former structures are indeed examples of VM is controversial, the accepted confirmation of VM presence is through the movement of injected tracers within tubular structures [73,74].

As may well be anticipated with a process that enables the survival and proliferation of a growing tumor, the process of EMT is speculated to be pivotal in VM [90]. A study showed 14% (28 of 205) of non-small cell lung cancer (NSCLC) tumors were classified as VM+ and statistically related to aggressive clinical course and poor prognosis [91]. In the same study, VM+ NSCLC samples displayed elevated levels of EMT-related proteins including vimentin, Slug, Twist, and stem-like proteins like nestin, CD44, MMP2 and MMP9. Accordingly, the epithelial marker E-cadherin was reduced in these samples, while VE-cadherin and β-catenin were elevated [91]. In breast cancer models the EMT associated transcription factor OCT4 is shown to correlate with VM, while Twist1 expression accelerates this process by increasing a population of CD133+ cells [92]. Ovarian cancers with evidence of VM also correlated with high expression levels of Twist1 and Slug [93]. In another study, ovarian cancer [85] and NSCLC [94] patients with VM+/CD133+ tumors predicted a poor prognosis; VE-cadherin, MMP-2 and MMP-9 were also reported to be increased in CD133+ xenografted tumors [82]. In hepatocellular carcinoma cells, the inhibition of E-cadherin degradation down-regulated VE-cadherin expression levels [95]. Again, in hepatocellular carcinoma cells, inhibition of the Rho-associated coiled coil-containing protein kinase 1 (ROCK1) attenuated an EMT expression profile and the process of VM [96]. Further studies are required to define whether VM is an exclusive property of metastasis initiating cells and if VM-capable circulating tumor cells (if indeed they exist) have a higher potential for metastasis.

Unsurprisingly, given the similarities with angiogenesis, genes implemented in VM are those previously associated with vascular (VE-cadherin, VEGFR 1 and 2, EphA2), embryonic (Nodal, Notch4), and hypoxia-related (hypoxia-inducible factor, Twist1) signaling pathways [97]. As with angiogenesis, an underlying mechanism of induction of VM seems to be hypoxia [73]. However, in vitro experiments show that this process can also occur in normoxia and certain authors have preferred to use the term hypoxia-accelerated tubular structure formation [93]. Further investigation is required to decipher whether hypoxia is an essential driving force behind VM. A further principal proangiogenic stimulus implemented in this process is the VEGF signaling pathway. Both VEGF and VEGFR2 have been implemented in VM [98,99]. However, this process has proven resilient in in vitro treatments with a range of angiogenesis inhibitors, including bevacizumab (Avastin, see Table 1) [100,101]. A subpopulation of melanoma cells that express the vascular cell adhesion molecule PECAM1, but not VEGFR2, have been reported to be capable of undergoing VM [102]. Re-introduction of AP-2α (a transcriptional repressor of PECAM1) in PECAM1+ cancer cells abolishes tube-forming ability, whereas AP-2α knockdown in PECAM1− tumor cells upregulates PECAM1 expression and promotes tube formation [102]. The ανβ5 integrin also correlated with VM and highly aggressive melanoma [102]. Ovarian VM+ tumors have higher expression of β-catenin and VEGF [86]. In hepatocellular carcinoma cells, VEGF-induced VM is also reported to involve myocyte enhancer factor 2C (MEF2C) together with β-catenin via the p38 MAPK and PKC signaling pathways [103]. The use of an inhibitor of PKCα also blocked the effects of Wnt5a enhanced vasculogenic capacity, motility and invasiveness of ovarian cancer cells [90]. Enhanced expression of Wnt5a was also correlated to VM in non-small cell lung cancer models [104]. In addition, in NSCLC tumors samples, the expression of Dickkopf-1, a negative regulator of the Wnt signaling pathway correlates with VM positivity [91]. Conversely, another study in colon cancer Wnt3a-overexpressing cells reports Dickkopf-1 correlates with VM negativity through decreases in VE-cadherin and VEGFR2 [105,106].

Lastly, many other proteins, including aguaporin 1 [107], have been postulated as essential regulators or promoters of VM, suggesting the search for the precise mechanism(s) and the unique pathways involved in this process is still very much in its infancy, mainly due to a lack of standardized, reliable in vitro models [108]. Future investigation and the identification of biomarkers may allow the specific targeting of the process of VM in the clinical practice.

## 5. Vessel (or Vascular) Co-Option

In addition to compensatory pathways and VM, cancer cells can overcome angiogenesis blockade through Vessel (or vascular) co-option (VCO). The term VCO is used to describe a process, by which cancer cells “hijack” preexisting blood vessels or capillaries to obtain their nutrient supply. Along with VM, VCO has been proposed to explain the lack of efficacy of antiangiogenic therapies and more recently, studies have also postulated a critical role of VCO in the establishment and survival of metastases. Initial studies in VCO hypothesized that cancer cells could arise or metastasize to highly vascularized tissues and co-opt the preexisting vasculature to enhance angiogenesis through a balance between VEGF and Angpt-2 [109], such studies also observed that VCO was followed by vessel regression, tumor hypoxia and the induction of angiogenesis by Angpt-1 and -2 and also VEGF [109]. Currently, VCO is recognized as a mechanism to generate tumor vasculature in an angiogenesis independent manner and despite being present in many malignancies it is more frequently seen in highly vascularized tissues such as brain [110,111], lungs [112] and liver [113] where cancer cells can co-opt the abundant pre-existing blood vessels and capillaries.

Clinical and experimental studies demonstrate that VCO is a common strategy used by cancer cells to evade antiangiogenic therapies; “normalization” of the vasculature by bevacizumab eliminates immature tumor microvessels, retaining the mature ones, in response to anti-VEGF treatment the remaining blood vessels increase their diameter, a response that also enhances VCO in colorectal cancer cells already metastasized to the liver [114]. In ovarian and esophageal cancer xenografts bevacizumab increases pericyte coverage of vessels [115], as mentioned previously pericytes play a major role in EC maintenance and resistance to VEGFR inhibitors. In a mouse model of pancreatic neuroendocrine tumors (PNETs), long-term treatment with a VEGFR2 blocking antibody generates resistant tumors with co-opted vessels with increased pericyte coverage [116] suggesting strategies that target pericytes (such as PDGFR inhibitors) could also be effective against VCO.

As stated, during the last decade a number of experimental studies have demonstrated VCO in many malignancies, however in most cases they use either murine models or xenografted cell lines. In contrast, studies using human tissues are rather limited [110,117,118]. One of the main obstacles to critically assess the contribution of VCO in patient samples is the identification of “angiogenic” versus non-angiogenic (pre-existing or mature) vessels. Experimental murine studies in the brain are performed by injecting cell lines in the carotid artery to induce infiltrative lesions in the brain parenchyma that exploit preexisting blood vessels, this is demonstrated by comparison with the normal brain vasculature assessing vessel diameter, endothelial activation and pericyte coverage [119]. Indeed, the presence of perivascular pericytes is commonly used as indicative of mature (preexisting) vessels that stain intensely with smooth muscle actin (SMA) compared to less mature (angiogenic) counterparts. LH39 is an antibody directed against an epitope of the basement membrane of normal capillaries and small venules; LH39 is more predominantly expressed in non-angiogenic (pre-existing) compared to angiogenic tumor vessels [120] and therefore might be a useful biomarker to discriminate between the two.

**Figure 3:**
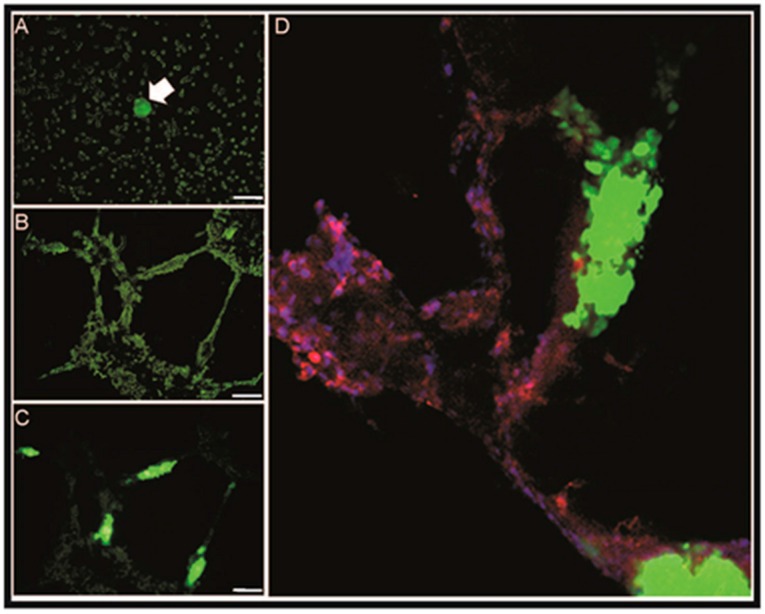
Vascular co-option in vitro model: (**A**) Merged brightfield-green fluorescent filter image of ECs (unlabeled) co-incubated on day 0 with a cancer sphere (marked with an arrow) composed of cells from the Ishikawa endometrial cancer cell line labeled with the green fluorescent protein ZsGreen; (**B**) merged image of cancer cells and EC tube formation at Day 5; (**C**) green filter image at Day 5 showing co-option (fluorescent cancer cells growing exclusively upon the tubular structures formed by the ECs in matrigel); and (**D**) zoom immunocytochemistry of EC (CD31+, stained red)-cancer cell (ZsGreen fluorescent) co-cultures. (Scale bars for A, B, C: 100 μm.)

In addition to xenografted cell lines, experimental VCO in vivo studies include a zebrafish model [121] and the chick chorioallantoic membrane (CAM) assay [122]. On the other hand, in vitro studies assessing VCO are more limited; Valiente et al. [111] uses fluorescently labeled breast cancer cell lines co-incubated with fresh mouse brain slices in organotypic cultures, in these conditions cancer cells migrate into the tissue, then seek and co-opt brain microcapillaries. In our laboratory, we have developed a co-culture system to assess VCO in vitro; ECs are grown in three-dimensional (3D) cultures on matrigel to form capillary-like structures and then co-incubated with fluorescently labeled cancer cells. Figure 3 shows that cancer cells (Zsgreen labeled) spread over capillary-like structures formed by ECs (CD31+, in red). Although our model lacks a basement membrane it clearly demonstrates that the direct interaction between capillary-like structures formed by ECs and cancer cells modifies cancer cell morphology. The study of the crosstalk between endothelial and cancer cells plays a crucial role in angiogenesis and metastasis [123]; our model recapitulates this interaction in a physiologically relevant context. Future studies in this interaction may bring new therapeutic targets to prevent VCO and metastasis.

Histologically, VCO can be defined as tumor cells spread along the external (abluminal) surface of vessels in a pericytic location with no intravasation, a biological phenomenon called Angiotropism [124] and a common feature of metastatic cancer cells. As mentioned above, the use of angiostatic drugs such as bevacizumab increases the diameter of preexistent (resistant or non-angiogenic) vessels and enhances VCO in cancer cells. Therefore, as suggested by others [125], it is possible that the suppression of angiogenesis, and the normalization of the vasculature might actually trigger angiotropism (VCO) and a more “metastatic” phenotype in cancer cells, accelerating metastasis. Indeed, the inhibition or suppression of angiogenic factors including VEGF, VEGFR2 [126,127,128,129], HIF1α [126,130] and VE-Cadherin [131] enhances an invasive behavior of tumors and VCO. This is further confirmed by a recent study that analyzed Sorafenib-resistant hepatocellular carcinomas and observed that these tumors were more locally infiltrative and co-opted liver vessel with a concomitant shift of cells to an EMT [132].

VCO is a characteristic feature of infiltrative, metastatic tumors; a study that selected subclones of breast cancer cell lines by their ability to metastasize to the brain demonstrated that these cells survive in the brain tissue by overexpressing Serpins, a class of Plasminogen Activator (PA) inhibitors that promote survival and VCO in brain metastasis [111], linking a metastatic phenotype in cancer cells with a “co-opting” behavior. Despite its key role in the establishment of micro-metastasis, the molecular basis for VCO in cancer is largely unknown. The above-mentioned study by Valiente et al. demonstrates that VCO in metastatic cells is dependent on L1CAM expression [111]. Another study that also analyzed brain metastases by breast cancer cells postulated VCO as crucial requisite for adhesion, proliferation and micro-colony establishment and identified Integrin β1 subunit in tumor cells as a critical mediator of these processes [110]. A third study used the CAM assay [122] and the orthotopic xenograft model of glioma [122,133] to demonstrate that selective inhibition of the inositol-requiring enzyme 1α (IRE1α) RNAse activity enhances the invasiveness and VCO in glioblastoma. Furthermore, IRE1α has a dual kinase/RNAse activity required for adaptive and stress responses (including ischemia) and a double (RNAse/kinase) inhibition generates reprogrammed cancer cells with a mesenchymal phenotype that produce avascular infiltrative glioblastomas with decreased MVD and enhanced VCO [133]. Paradoxically, the survival rates in these animals are increased compared to animals with a functional IRE1 [122]. The molecular pathways involved and the contribution of the above mentioned factors to VCO in other target tissues for metastases such as lungs and the liver remain to be elucidated.

As above-mentioned many studies suggest that angiogenesis and VCO (expressed as invasiveness) are functionally related mechanisms, however they seem mutually exclusive phenomena in cancer cells. The use of angiogenesis inhibitors might cause an adaptive switch into an invasive, more metastatic phenotype of cancer cells resembling EMT; therefore, we speculate that second-line therapies that target VCO might help patients with antiangiogenic resistant tumors.

## 6. Future Directions for Antiangiogenic Therapies

**Figure 4:**
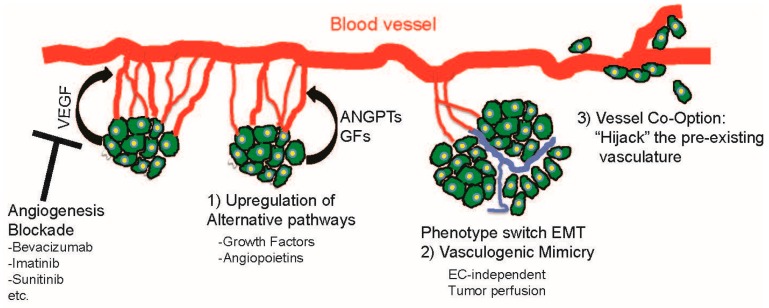
Strategies employed by cancer cells to escape antiangiogenesis. Summary of the strategies reviewed in this article. Growing tumors require a blood supply, which is obtained by the production of VEGF that stimulates angiogenesis. Most antiangiogenic compounds target the VEGF pathway (Angiogenesis Blockade). However, tumors can overcome angiogenesis blockade by upregulation of alternative pathways such as angiopoietins or growth factors. In addition, a subset of cells within the tumor can switch to an epithelial-to-mesenchymal transition (EMT) phenotype establishing a tumor perfusion system within a tumor; independent of endothelial cells a process known as vasculogenic mimicry. Lastly, tumor cells can obtain their blood supply by “hijacking” pre-existent blood vessel through vessel co-option.

In recent years, the widespread use of therapeutic drugs targeting angiogenesis has had a major impact in cancer patients. However, in many cases despite a favorable initial response, the patients relapse and an increase in overall patient survival is either minimal or not significant [134]. Today most antiangiogenic regimes used in the clinic target the VEGF pathway (either ligands or receptors, Figure 4). As any biological system that adapts to a selective pressure, cancer cells display a variety of strategies to overcome the blockade of angiogenesis and obtain access to nutrients and oxygen. Independent inhibition of the VEGF and angiopoietin pathways has demonstrated clinically meaningful prolongation in progression-free survival in front line settings [59]. A future strategy may be simultaneous dual targeting of angiogenesis and alternative/compensatory angiogenic escape route. If the obvious problems of enhanced toxicity in the presence of dual agents can be overcome, this method may reduce the changes of angiogenic escape and possible move antiangiogenic therapies into the front line of cancer treatments, apposed to their current use in the adjuvant setting. In this context, optimized or even targeted drug delivery with reduced doses (lowering toxicity) using nano-encapsulated drugs may offer a viable choice; in fact, a recent study used IM-loaded nanoparticles and demonstrated lower cardiotoxicity in rats and improved anticancer activity in breast cancer cells [135]. In addition, the use of combined drugs with synergistic effects in angiogenesis blockade should be explored within this context.

Recurrent metastatic disease with prolonged latency periods is speculated to result from reactivation of “dormant” cancer cells [136]. Here, cancer cells or a small group of metastatic cancer cells may also experience angiogenic dormancy and thus survive by receiving their nutrients and oxygen from VCO. Therefore, the clinical observation where residual disease is present but remains asymptomatic, may involve dormant cancer cells incorporating many characteristics that are present in antiangiogenic therapy resistant tumors. This gives rise to the possibility that therapeutic targeting of antiangiogenic resistant tumors could also allow selective targeting of residual disease.

Regarding VM several treatments have been proposed: Liposomes containing epirubicin plus celecoxib and liposomes containing a peptide motif (that targets aminopeptidase) have been suggested to reduce VM in brain gliomas. While these constructs may have activity over tumor growth, the evidence is still unconvincing that VM is a target of these therapeutic constructs [137,138]. Growing evidence suggest targeting of the Wnt/β-catenin pathway, this might be a viable treatment modality against VM having both antiangiogenic and antitumorigenic effects. As is now the standard in modern oncology, any potential new treatment will require a biomarker or companion for diagnostic in order to recommend their use. As stated, the mechanism(s) behind VM are still poorly understood, however when these specific pathways are elucidated the use of precision medicine directed against overexpressed and/or mutated oncogenes will have a double effect in reducing tumor size and its ability to undergo VM. The ability of only a certain percentage of tumors to undergo VM raises important clinical questions as we enter the circulating tumor cell (CTC) detection era. Is there an intrinsic ability of a CTC to undergo VM once a metastatic niche has been found or established? Does this ability determine the location of the eventual metastasis? Moreover, can VM potential be screened for using current CTC technology? While most of these questions remain open, a recent paper suggests a correlation between VM and the metastatic niche [139]. In prostate cancer dual CD31/PAS staining shows 35% (28/80) of cases were VM+ and exhibit poor patient prognosis. The incidence of bone metastasis in VM+ and VM− patients was 68% (19/28) and 39% (20/52), respectively, suggesting VM+ prostate cancers are more prone to develop bone metastasis. This may be a key finding for the future of CTC characterization and design of anti-vascular drugs.

VCO is not only an antiangiogenic therapy resistance mechanism but also a key factor in the establishment of micrometastases [111]. The majority of cancers metastasize to highly vascularized organs (brain, liver and lungs), a process that requires, at least initially co-opting the pre-existing vasculature to obtain a blood supply, therefore the same factors that mediate metastatic spread to these organs could also be important determinants for VCO. Surprisingly, the molecular bases of VCO in cancer cells are largely unknown; as occurs with VM+ cells, cancer cells that exhibit an enhanced VCO behavior are also characterized by an EMT phenotype. Among potential therapeutic targets all the above mentioned factors have already been explored; bioinformatics demonstrate L1CAM gene is a miR503 (microRNA) target, miR503 overexpression reduces cell proliferation and invasion of glioma cells, suggesting it could be used as a L1CAM negative regulator/suppressor [140]. Similarly, integrin β1 subunit, another potential therapeutic target against VCO, can be downregulated by miR-223 in prostate cancer cells [141].

In summary, cancer cells display a wide variety of mechanisms to overcome angiogenic blockade, and these are not exclusively limited to those reviewed herein (summarized in Figure 4). A recent study points to other areas of angiogenesis that could be exploited for therapeutic use, namely: EC migration, structural abnormalities of tumor vessels, hypoxia, lymphangiogenesis, elevated interstitial fluid pressure, poor perfusion, disrupted circadian rhythms, tumor promoting inflammation, tumor promoting fibroblasts and tumor cell metabolism/acidosis [142]. If toxicity issues permit, future therapies should be combined to target key aspects that are specific for tumor angiogenesis. Finally, the arrival of personalized (or precision) medicine may shed some light on the angiogenic mechanisms employed by the tumor mass, such as information on the presence of VM, VCO and tumor metabolism (among many other possible variables) needed to complement the levels of proangiogenic factors such as angiopoietin or VEGF. Thus, along with de novo drug discovery, there is a clinical demand in order to reveal effective biomarkers that will identify pathways and predict patient outcomes in response to antiangiogenic compounds.

## Figures and Tables

**Table 1:** Antiangiogenic compounds currently approved by the USA Food and Drug Administration (FDA).

Drug Name (Commercial)	Cancer Type (Recommended Use)	Main Mechanism of Action
Bevacizumab (Avastin)	Metastatic colorectal	VEGF antibody
	Renal cell carcinoma (RCC)	
	Non-small cell lung cancer (NSCLC)	
	Ovarian	
Ramucirumab (Cyramza)	Advanced stomach	VEGFR2 antibody
	Gastroesophageal adenocarcinoma	
	NSCLC	
	Metastatic colorectal	
Cetuximab (Erbitux)	CRC	EGFR antibody
	Head and neck squamous cell carcinoma	
	NSCLC	
Panitumumab (Vectibix)	CRC	EGFR antibody
Trastuzumab (Herceptin)	HER2+ breast cancer	EGFR antibody
Aflibercept (Zaltrap or Eylea)	Colorectal cancer (CRC)	VEGF-trap *
Sunitinib (Sotent)	RCC	TKI
	Pancreatic neuroendocrine tumors (PNETs)	
	Gastrointestinal stromal tumors (GISTs)	
Axitinib (Inlyta)	RCC	TKI
Vandetanib (Caprelsa)	Medullary carcinoma of thyroid	TKI
Lenvatinib (Lenvima)	Thyroid	TKI
Pazopanib (Votrient)	RCC	TKI
	Soft tissue sarcoma	
Cabozantinib (Cometriq)	Medullary thyroid cancer	TKI
Erlotinib (Tarceva)	Pancreatic cancer	TKI
Gefitinib (Iressa)	Lung cancer	TKI
	Breast cancer	
Imatinib (Glivec)	Chronic myeloid leukemia	TKI
	Acute lymphoid leukemia	
	GISTs	
Lapatinib (Tyverb)	HER2+ breast cancer	TKI
Sorafenib (Nexavar)	Hepatocellular carcinoma	TKI
	RCC	
	Thyroid	
Regorafenib (Stivarga)	Refractory metastatic colorectal	TKI
	Advanced GISTs	
Thalidomide (Thalomid)	Multiple myeloma	Immunomodulator
Lenalidomide (Revlimid)	Multiple myeloma	Immunomodulator
	Non-Hodgkin lymphoma	
Rapamycin (Sirolimus)	RCC	mTOR inhibitor
Temsirolimus (Torisel)	RCC	mTOR inhibitor
Everolimus (Afinitor)	RCC	mTOR inhibitor
	Advanced breast	
	PNETs	

Abbreviations: TKI, tyrosine kinase inhibitor; mTOR, mammalian target of rapamycin. * For a full description of VEGF-trap see text.
